# Obtaining insights from high-dimensional data: sparse principal covariates regression

**DOI:** 10.1186/s12859-018-2114-5

**Published:** 2018-03-27

**Authors:** Katrijn Van Deun, Elise A. V. Crompvoets, Eva Ceulemans

**Affiliations:** 1 Department of Methodology & Statistics, Tilburg University, Warandelaan 2, Tilburg, 5000 LE The Netherlands; 2 Department of Psychology, KU Leuven, Tiensestraat 102, Leuven, 3000 Belgium

**Keywords:** Dimension reduction, Prediction, High-dimensional data, Immunology, Stability selection

## Abstract

**Background:**

Data analysis methods are usually subdivided in two distinct classes: There are methods for prediction and there are methods for exploration. In practice, however, there often is a need to learn from the data in both ways. For example, when predicting the antibody titers a few weeks after vaccination on the basis of genomewide mRNA transcription rates, also mechanistic insights about the effect of vaccinations on the immune system are sought. Principal covariates regression (PCovR) is a method that combines both purposes. Yet, it misses insightful representations of the data as these include all the variables.

**Results:**

Here, we propose a sparse extension of principal covariates regression such that the resulting solutions are based on an automatically selected subset of the variables. Our method is shown to outperform competing methods like sparse principal components regression and sparse partial least squares in a simulation study. Furthermore good performance of the method is illustrated on publicly available data including antibody titers and genomewide transcription rates for subjects vaccinated against the flu: the selected genes by sparse PCovR are higly enriched for immune related terms and the method predicts the titers for an independent test sample well. In comparison, no significantly enriched terms were found for the genes selected by sparse partial least squares and out-of-sample prediction was worse.

**Conclusions:**

Sparse principal covariates regression is a promising and competitive tool for obtaining insights from high-dimensional data.

**Availability:**

The source code implementing our proposed method is available from GitHub, together with all scripts used to extract, pre-process, analyze, and post-process the data: https://github.com/katrijnvandeun/SPCovR.

## Background

Traditionally, data analysis methods are divided in two classes with different goals: Methods for prediction (or, supervised learning) and methods for exploration (or, unsupervised learning). An example of the former is assessing whether someone is at risk for breast cancer; in this case the aim is to use currently available information to predict an unseen (often future) outcome. On the other hand, the goal of exploratory methods is to gain an understanding about the mechanisms that cause structural variation and covariation in the available information. For example, exploration of gene expression data collected over time after addition of serum gave not only insight in the transcriptional program but also in processes related to wound repair [1]. There are many cases, however, where it is of interest to reach both objectives and to predict an outcome of interest while simultaneously revealing the processes at play. This is for example the case in the study of [2]: The gene expression response soon after vaccination and the antibody titers much later in time were measured with the aim of both predicting immunogenicity and revealing new mechanistic insights about vaccines.

To reveal the underlying mechanisms, component or matrix decomposition based methods can be used. Well known examples are principal component analysis (PCA) and the singular value decomposition [3]. Yet, another frequent use of such methods is in the context of prediction with many covariates: A popular approach is to first reduce the covariates to a limited number of components and to subsequently use these for prediction. This is known as principal components regression (PCR, see [4]). A drawback of this two-step approach is that the components are constructed with no account of the prediction problem and hence may miss the components that are relevant in predicting the outcome. This is especially true when the number of predictor variables is huge and represents a large diversity of processes, as is for example the case with genomewide expression data. Sparse regression approaches like the lasso [5] and elastic net [6], on the other hand, only focus on modeling the outcome with no account of the structural variation underlying the covariates. Hence, approaches that find components that simultaneously reveal the underlying mechanisms and model the outcome of interest are needed. Partial least squares (PLS; see for example [7]) and principal covariates regression, PCovR [8], are such methods. Yet, partial least squares may have a too strong focus towards prediction [9] while principal covariates regression can be flexibly tuned to balance between prediction of the outcome and reduction of the covariates to a few components.

Apart from implementation issues (meaning that existing PCovR software can only be used on data with a modest number of variables), a shortcoming of PCovR is that the components are based on a linear combination of all variables. This is undesirable when working with a large set of variables, both from a statistical and an interpretational point of view. First, the estimators are not consistent in the *p*>*n* case [10], and second, the interpretation of components based on a high number of variables is infeasible. Furthermore, components that are based on a limited set of selected variables better reflect the fact that many biological processes are governed by a few genes only. To overcome such issues in partial least squares, sparse methods have been developed [10, 11]. Likewise we propose here a sparse and efficient version of principal covariates regression^1^. The proposed method offers a flexible and promising alternative to sparse partial least squares.

The paper is organized as follows. First we propose the PCovR method and its sparse extension (SPCovR), and we discuss its relation to (sparse) PLS. The (comparative) performance of SPCovR is evaluated in a simulation study and in an application to genomewide expression data collected for persons vaccinated against the flu [2]. The implementation of the SPCovR algorithm is available online (https://github.com/katrijnvandeun/SPCovR) together with the scripts used for analyzing the data.

## Methods

### Sparse principal covariates regression

We will make use of the following notation: matrices are denoted by bold uppercases, the transpose by the superscript ^*T*^, vectors by bold lowercase, and scalars by lowercase italics. Furthermore, we will use the convention to indicate the cardinality of a running index by the capital of the letter used to run the index (e.g., this paper deals with *J* variables with *j* running from 1 to *J*), see [12].

#### Formal model

The data consist of a block of predictor variables **X** and a block of outcome variables **Y**. We will assume all variables to be centered and scaled to sum of squares equal to one. Now, consider the following decomposition of the *I*×*J*_*x*_ matrix of covariates **X**,

1 $$ \mathbf{X}=\mathbf{XWP}_{x}^{T}+\mathbf{E}_{x}=\mathbf{TP}_{x}^{T}+\mathbf{E}_{x},  $$

together with the following rule to predict the *J*_*y*_ outcome variables **Y**,

2 $$ \mathbf{Y}=\mathbf{XWP}_{y}^{T}+\mathbf{E}_{y}=\mathbf{TP}_{y}^{T}+\mathbf{E}_{y},  $$

with **XW**=**T** the *I*×*R* matrix of component scores, **P**_*x*_ the *J*_*x*_×*R* matrix of variable loadings, **P**_*y*_ the *J*_*y*_×*R* vector of *R* regression weights, and **E**_*x*_, **E**_*y*_ the residuals. Note that we consider the general problem of a multivariate outcome, hence *R* regression coefficients for each of the *J*_*y*_ outcome variables. The component scores are often constrained to be orthogonal : **T**^*T*^**T**=**I** with **I** an identity matrix of size *R*×*R*. Despite this restriction there still is rotational freedom and the PCovR coefficients are not uniquely defined. The data model represented in (1) and (2) is one that summarizes the predictor variables by means of a few components and uses these as the predictors of the outcome. Note that the same model also underlies principal components regression and partial least squares.

#### Objective function

Principal covariates regression [8] differs from the former methods in the objective function used: Minimize over **W,P**_*x*_,**P**_*y*_

3 $$\begin{array}{@{}rcl@{}} L(\mathbf{W,P}_{x},\mathbf{P}_{y}) &=& (\!1\,-\,\alpha) \frac{\| \mathbf{Y}\,-\,\mathbf{XWP}_{y}^{T} \|^{2}}{\|\mathbf{Y}\|^{2}}+ \alpha \frac{\| \mathbf{X}-\mathbf{XWP}_{x}^{T}\|^{2}}{{\|\mathbf{X}\|^{2}}} \\ &=& \left\| \left[ w_{1} \mathbf{Y} \ w_{2}\mathbf{X} \right]-\mathbf{XW} \left[ w_{1}\mathbf{P}_{y}^{T} \ w_{2}\mathbf{P}_{x}^{T} \right] \right\|^{2}  \\ &=& \left\| \mathbf{Z} - \mathbf{XWP}^{T} \right\|^{2} \end{array} $$

such that **T**^*T*^**T**=**I** and with 0≤*α*≤1, $w_{1}=\sqrt {1-\alpha } \slash \|\mathbf {Y}\|$, $w_{2}=\sqrt {\alpha } \slash \|\mathbf {X}\|$, **Z**=[*w*_1_**Y** *w*_2_**X**], and $\mathbf {P}=\left [ w_{1}\mathbf {P}_{y}^{T} \ w_{2}\mathbf {P}_{x}^{T} \right ]^{T}$. The parameter *α* is a tuning parameter giving either more weight to the prediction of the outcome (*α* close to 0) or to the reconstruction of the predictor variables (*α* close to one). In fact, *α*=1 corresponds to principal components regression while *α*=0 corresponds to ordinary regression. Let $R_{X}^{2}$ denote the percentage of variance in **X** accounted for by **T** and $R_{Y}^{2}$ the percentage of variance in **Y**. It can be seen then that the criterion is equivalent to maximizing

4 $$ \alpha R_{X}^{2}+(1-\alpha)R_{Y}^{2}.  $$

A solution to (3) based on the singular value decomposition of **X** was proposed by [13]. An efficient implementation that accounts for large data (either *I* or *J* large) can be found in the online code.

Partial least squares is based on the optimization of the following criterion [8, 10]

5 $$ \mathbf{w}_{r}=\arg \,\max_{\mathbf{w}}\mathbf{w}_{r}^{T}\mathbf{X}^{T}\mathbf{Y}\mathbf{Y}^{T}\mathbf{X}\mathbf{w}_{r}  $$

for *r*=1,…,*R* and such that $\mathbf {w}_{r}^{T}\mathbf {w}_{r}=1$ for all *r*=1,…,*R* and $\mathbf {w}_{r}^{T}\mathbf {X}^{T}\mathbf {Xw}_{r'}=0$ for *r*≠*r*^′^. Note that this is equivalent to maximizing

6 $$ \text{var}(\mathbf{Xw}_{r})\text{corr}^{2}(\mathbf{Xw}_{r},\mathbf{Y})  $$

under the restrictions. Criterion (6) is approximately equal to $R_{X}^{2}R_{Y}^{2}$ and can be compared to criterion (4) to obtain some intuition about the similarities and differences between both methods. Given that the PLS and PCovR criteria are different, it can be expected that the obtained estimates are different as well. Whereas PLS cannot be expressed as a special case of PCovR with a particular value of the tuning parameter *α*, it has been shown to be a special case of continuum regression with the continuum regression parameter set equal to 0.5 [14].

A drawback of the principal covariates regression model is that the components are based on a linear combination of all the predictor variables. Having components that are characterized by a few variables only is easier to interpret and often a better reflection of biological principles. This motivates the introduction of a sparseness restriction on the component weights *w*_*jr*_:

7 $$\begin{array}{@{}rcl@{}} L(\mathbf{W,P}_{x},\mathbf{P}_{y}) &=& (1\,-\,\alpha) \frac{\| \mathbf{Y}-\mathbf{XWP}_{y}^{T} \|^{2}}{\|\mathbf{Y}\|^{2}}\,+\,\alpha \frac{\| \mathbf{X}\,-\,\mathbf{XWP}_{x}^{T}\|^{2}}{{\|\mathbf{X}\|^{2}}} \\ && +\lambda_{1}|\mathbf{W}|_{1} +\lambda_{2}|\mathbf{W}|_{2}^{2} \end{array} $$

with $|\mathbf {W}|_{1}=\sum _{j,r} |w_{jr}|$ the lasso penalty and $|\mathbf {W}|_{2}^{2}=\sum _{j,r} w_{jr}^{2}$ the ridge penalty. *λ*_1_ (*λ*_1_≥0) and *λ*_2_ (*λ*_2_≥0) are tuning parameters for the lasso and ridge penalties respectively. The effect of the lasso is that it shrinks the coefficients, some (or many for high *λ*_1_) to exactly zero thus performing variable selection. Note that the lasso penalty in Eq. (7) is imposed only on the component weights and not on the loadings **P**_*x*_ nor on the regression weights **P**_*y*_. The penalty implies that some or many of the component weights will become zero; because the loadings and regression weights are not subject to the lasso penalty, these are not affected by the penalty. The ridge also introduces shrinkage and is included here for two reasons: To introduce stability in the estimated coefficients and to allow for more than *I* non-zero coefficients; this combination of penalties is known as the elastic net. Both the lasso and the elastic net are known to over-shrink the non-zero coefficients [15, 16]. One way to undo the shrinkage of the non-zero coefficients, is to re-estimate them using an ordinary least squares approach [17]. When *α*=1, the objective function (7) reduces to the sparse PCA criterion [18] and the resulting estimates can also be obtained under a sparse principal components regression approach. When *α*=0 and *R*=1, the elastic net regression formulation is obtained [6] and the two problems are equivalent. Note that the introduction of the sparseness restriction eliminates the rotational freedom and, under suitable conditions, has a unique solution.

Similarly, sparse PLS approaches have been proposed that are based on the same penalties:

8 $$ \arg\,\max_{\mathbf{w}} \quad \mathbf{w}_{r}^{T}\mathbf{X}^{T}\mathbf{Y}\mathbf{Y}^{T}\mathbf{X}\mathbf{w}_{r} +\lambda_{1}|\mathbf{w}_{r}|_{1} +\lambda_{2}|\mathbf{w}_{r}|_{2}^{2}.  $$

This sparse PLS criterion is different from the SPCovR criterion (7) over the whole range of *α*. The two methods can be expected to yield different estimates.

#### Algorithm

The procedure that we will use to estimate the model parameters is one which estimates all *R* components simultaneously and not -as is often the case in the literature - one by one. The main benefit is that this gives control over the constraints that are imposed on the parameter estimates. More specifically, we offer the choice to constrain the loadings **P**_*x*_ either to be orthogonal or length restricted ($diag \left (\mathbf {P}_{x}^{T}\mathbf {P}_{x}\right)=\mathbf {1}$). The former is the usual constraint used in sparse PCA approaches [18], the latter is more flexible and allows for correlated component loadings. Note that the length constraint is needed to avoid trivial solutions where very small component weights that satisfy the penalty are compensated by very high loadings. To solve the optimization problem in (7) under these constraints, we rely on a numerical procedure and alternate between conditional estimation of **W** given fixed values for **P** and of **P** given fixed values for **W**. For the moment we assume the number of components *R* and the value of the tuning parameters *α*, *λ*_1_, and *λ*_2_ to be given; how to tune these meta-parameters is discussed in the next subsection.

The conditional estimation of the weights **W** is based on a coordinate descent procedure and of the loadings on a restricted least-squares routine; both procedures are detailed in the Appendix. Using these routines, the loss is guaranteed to be non-increasing. Furthermore, because the loss is bounded from below by zero the algorithm converges to a stationary point (for suitable starting values). To deal with the problem of local optima, a multistart procedure can be used. We recommend to use a combination of both a rational and several random starting configurations. A rational start may be obtained from the non-sparse principal covariates regression analysis. Note that often sparse multivariate approaches are initialized by a rational start only which does not account for the problem of local optima.



#### Tuning and model selection

The sparse PCovR model is estimated with fixed values for the weighting parameter *α*, the number of components *R*, the Lasso tuning parameter *λ*_1_, and the ridge parameter *λ*_2_. The problem that we consider here, is how to tune these meta-parameters. Cross-validation is frequently recommended in the literature but this requires data that are rich in the number of observations. In addition, the computational cost of cross-validation for the SPCovR model is considerable (because all possible combinations of the values for each of the tuning parameters need to be considered). Furthermore, in the context of PCovR, simulation studies showed that this is not a superior model selection strategy compared to strategies that rely on a stepwise approach [9]. Hence, we propose to use a stepwise strategy.

First, *α* is determined using the so-called maximum likelihood approach [19]:

9 $$ \alpha = \frac{J_{x}}{J_{x}+J_{y}\frac{\sigma_{\epsilon_{x}}^{2}}{\sigma_{\epsilon_{y}}^{2}}},  $$

with $\sigma _{\epsilon _{x}}^{2}$ and $\sigma _{\epsilon _{y}}^{2}$ the variance of the error on the predictor and outcome variables respectively. In the case of a large number of predictor variables *J*_*x*_ will dominate the expression and we can assume that *α* will be almost - but not exactly - equal to one without having to estimate the size of the error variances. It is important to keep *α* strictly smaller than one, for example *α*=.99, and to use PCovR instead of a PCR approach [19].

Second, we fix the number of components by a so-called scree test that is based on a visual display of the value of the loss function (3) against the number of components *r* in the model for *r*=1,...,*R*. In this display, we look for the point where the plot levels off and select the number of components just before this point.

Next we tune the ridge penalty. We recommend to set *λ*_2_ equal to a small value to have more emphasis on variable selection by the lasso (for example, 5% of the value of the lasso). This small value should be sufficient to stabilize the estimates and to encourage grouping of strongly correlated variables [20].



The final metaparameter to tune is the lasso parameter *λ*_1_. A straightforward and often used procedure to find a proper value for *λ*_1_ is cross-validation [6]. In the more recent literature it has been established that cross-validation results in selecting a superset of the correct predictors, and thus in false positives (see for example the retrospective paper on the lasso and the included discussions [21]). One proposal to address this issue of falsely selecting variables, is the use of a stability selection procedure [22] which allows to control the false positive error rate. Stability selection is a general method that can be easily applied (in adapted form) with our SPCovR procedure. In brief, it is a resampling based procedure that is used to determine the status of the coefficients (zero or non-zero) over a range of values for the tuning parameter *λ*_1_. The original procedure has been proposed for a single set of variable coefficients. Here, we have *R* such sets due to the fact that we estimate the weights of all components simultaneously. The order of the components between different solutions may change (permutational freedom of the components) and this has to be taken into account.

To explain the stability selection procedure, we start with the for loop in Algorithm 2: Given a fixed value *λ*_1_, *N* resamples are created by drawing with replacement a fraction *f* of the observations (with.50≤*f*<1). The resampled data are subjected to a SPCovR analysis and the resulting *N* matrices of component weights **W** are used as follows: For each coefficient *w*_*jr*_ the proportion of occurences for which it is non-zero in the *N* resamples is recorded in the probability matrix **Π**^(*λ*)^. Note that between samples, permutation of the components may occur. We account for this by permuting the components to maximal agreement in the component scores as measured by Tucker’s coefficient of congruence; the component score matrix resulting from the SPCovR analysis of the original (non-resampled) data is used as the reference for congruence.

Next, we turn to the while loop in which *λ*_1_ is decreased until the condition *q*_*Λ*_>*q*_*R*_ is met with *q*_*Λ*_ the number of non-zero coefficients over the range of *λ*_1_ values considered so far and *q*_*R*_ a value that results from controlling the expected number of falsely non-zero coefficients *V*. From [22] we have that the expected number of non-zero coefficients *q* is

$$ E(V) \leq \frac{1}{2\pi_{thr}-1}\frac{q^{2}}{J}   $$

or,

10 $$ q \leq \sqrt{J(2\pi_{thr}-1)E(V)}.  $$

Note that this is the expression for a single component; to obtain the upperbound *q*_*R*_ for *R* components we use

11 $$ q_{R} \leq R\sqrt{J(2\pi_{thr}-1)E(V)}.  $$

Hence, by fixing *E*(*V*), e.g. to one, and the probability threshold *π*_*thr*_=0.90 [22], an upper bound on the number of non-zero coefficients is obtained. For a range *Λ* of *λ*_1_ values, the non-zero probability is given by **Π**^(*Stable*)^= max*λ*∈*Λ***Π**^(*λ*)^ and the set of non-zero coefficients by those for which $\pi _{jr}^{(Stable)}\geq \pi _{thr}$. If *q*_*Λ*_≤*q*_*R*_ the procedure continues by extending the range of *λ*_1_ values with the next value. The values of *λ*_1_ are taken from the interval *Λ*=[ *λ*_*max*_,...,*λ*_*min*_] with *λ*_*min*_=1*e*^−4^*λ*_*max*_ and the remaining values equally spaced and arranged in decreasing order between log_2_(*λ*_*max*_) and log_2_(*λ*_*min*_); see [23].

## Results

To compare the performance of sparse principal covariates regression with competing methods, we make use of both synthetically created data in a simulation study and empirical data resulting from a systems biology study on the flu vaccine.

### Simulation study

In a first study on the performance of SPCovR, we make use of artificially generated data. The main aim is to study the behavior of SPCovR, also in relation to competing methods, in function of the strength of the components in relation to the covariates on the one hand and in relation to the outcome on the other hand. Therefore, the following factors were chosen to set up the simulation experiment based on a model with two components (see [9] for a similar setup):

1. VAFX: The total proportion of variation accounted for (VAF) by the components in the block of covariates with levels 0.01, 0.40, and 0.70.
2. The relative strength of the components in the variation accounted for in the block of covariates VAFX: 0.10 versus 0.90 (the second component is much stronger than the first component; for example with VAFX =0.40 the first component accounts for 4 percent of the total variation and the second one for 36 percent), 0.50 versus 0.50 (equally strong), and 0.90 versus 0.10.
3. VAFY: The total proportion of variation accounted for by the components in the outcome with levels 0.02, 0.50, and 0.80.

All factors were crossed, resulting in a simulation experiment with 3×3×3=27 conditions. The number of observations and variables was fixed to *I*=100 and *J*=200 respectively, 80% of the component weight coefficients were set equal to zero (this is 320 of the in total 400 coefficients), and the regression weights for the first and second component were set equal to *b*_1_=1 and *b*_2_=−0.02, implying that the first component is much more associated to the outcome than the second one (for equally strong components).

We expect SPCR to perform well - in terms of recovering the components - in all conditions where the components account for a considerable amount of variation in the covariates (VAFX = 0.40/0.70) but not when the components are submerged in the noise (VAFX = 0.01). In terms of prediction, SPCR can be expected to perform well when the components not only account for variation in the covariates but also in the outcome; when VAFY = 0.02 predictive performance can be expected to be bad for any method, including SPCR. For SPLS, we expect good performance when the components account for quite some variation both in the covariates and the outcome (VAFX = 0.40/0.70 and VAFY = 0.50/0.80) but poor performance when eiter VAFX or VAFY is low. Lastly, we expect SPCovR to perform well in terms of recovering the components when either VAFX or VAFY is considerable but not when both are low (VAFX = 0.01 and VAFY = 0.02). In terms of prediction, performance of SPCovR will be bad when VAFY = 0.02.

To generate data under a sparse covariates regression model with orthogonal loadings, the setup briefly described here was used. Full details can be found in the online available implementation: https://github.com/katrijnvandeun/SPCovR. An initial set of weights **W**^(0)^ was obtained by taking the first two right singular vectors obtained from an *I*×*J* matrix **X**^(0)^ generated by random draws from a standard normal distribution. Sparsity was created by setting 320 values, chosen at random, to zero. Next, the resulting sparse weight vector was rescaled according to the relative strength of the components in the condition considered. These initial component weights **W**^(0)^ and the fixed regression weights *b*_1_=1 and *b*_2_=−0.02 were used to calculate an initial outcome vector,

12 $$ \mathbf{y}^{(0)}=\mathbf{X}^{(0)}\mathbf{W}^{(0)} \left\lbrack \begin{array}{c} 1 \\ -0.02 \end{array}\right\rbrack.  $$

Note that the initial matrix **X**^(0)^ was not generated under a SPCovR model. To obtain data that perfectly fit such a model, a principal covariates regression analysis with fixed zero weights was performed to yield sparse component weights **W** and orthogonal loadings **P**. Again, the weights were rescaled and a block of covariates **X**^*TRUE*^=**X**^(0)^**WP**^*T*^ and the outcome **y**^*TRUE*^=**X**^*TRUE*^**W**[*b*_1_*b*_2_]^*T*^ were calculated on the basis of the scaled component weights and the loadings resulting from the SPCovR analysis. These are data with no noise and in a final step noise was added by sampling from the normal distribution with mean zero and variance set in correspondence to the level of the proportion of variation accounted for by the components in the covariates and the outcome yielding data **X** and **y**. For each of the 27 conditions, 20 replicate data sets were generated resulting in 540 data sets in total. The code used to generate the data, including the seed used to initialize the pseudo random number generator, can be found on GitHub: https://github.com/katrijnvandeun/SPCovR.

Each dataset was subjected to five analyses: sparse principal components regression (SPCR), sparse partial least squares (SPLS), and three SPCovR analyses with different values of *α*, namely 0.01, 0.50, and 0.99. For the SPCR analysis, we used the elasticnet R package that implements the sparse PCA approach in [18]. The elasticnet R package uses a least angle regression (LARS) [17] procedure and hence allows to find a solution with a defined number of zeros for each of the components. We set this number equal to the exact number of zero coefficients occuring in **W**. For the SPLS analysis, the R package RGCCA was used that allows to (approximately) set the total number of zero coefficients over the components; the analyses options were set to result in (approximately) 320 zero coefficients. For the SPCovR analyses, we used stability selection with the upperbound on the number of non-zero coefficients *q* set equal to 80. Hence all analyses were tuned such that they had exactly or approximately the same number of zeroes as present in the true underlying component weight matrix. This makes the interpretation of the resuls easier in the sense that performance of the methods is not dependent upon proper tuning of the sparseness penalty. In the comparison of the methods, we will consider their performance in recovering the underlying components and how well they predict a new set of test data (generated under the same model, this is with the same **W**, regression weights, and the same error level for the covariates and outcome).

**Fig. 1:**
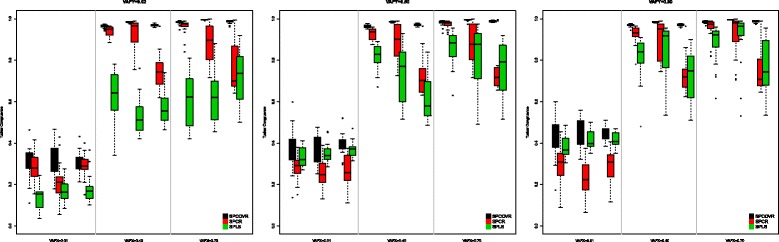
Tucker congruence for the simulated data

The results with respect to the recovery of the components is shown in Fig. 1. These boxplots display the Tucker congruence between the true componentscores **T**=**X**^*TRUE*^**W** and those obtained from the analyses $\hat {\mathbf {T}}=\mathbf {X}\hat {\mathbf {W}}$. Tucker congruence, *ϕ*, is defined as [24],

13 $$ \phi = \frac{\text{vec} (\mathbf{T})^{T}\text{vec} \big(\hat{\mathbf{T}}\big)} {\sqrt{\left(\text{vec} (\mathbf{T})^{T} \text{vec} (\mathbf{T}) \right) \left(\text{vec} \big(\hat{\mathbf{T}}\big)^{T} \text{vec} \big(\hat{\mathbf{T}}\big) \right)}}  $$

this is the cosine of the angle between the vectors vec(**T**) and $\text {vec} (\hat {\mathbf {T}})$ with higher values indicating more similarity between the components. Values over 0.95 indicate that the components can be considered equal while values in the range [ 0.85−0.94] correspond to a fair similarity [24]. In Fig. 1 the Tucker congruence of the 20 replicate data sets is shown for the 27 conditions and the three methods (SPCovR with *α*=0.99, SPCR, and SPLS). For each combination of the variation accounted for in the covariates and in the outcome (e.g., VAFX = 0.01 and VAFY = 0.02 at the left of the left panel), three boxplots are shown for the three methods. These are the three levels of the relative strength factor with the boxplots at the left referring to the conditions where the first component is weaker than the second one, the boxplots in the middle referring to the conditions where they are equally strong, and boxplots at the right to the conditions where the second component is stronger than the first one. SPCovR outperforms the two other methods in all conditions, followed by SPCR which outperforms SPLS in most conditions. Only when the variance accounted for by the components in the block of covariates (the conditions VAFX = 0.01) is very low while it is high in the outcome variable (VAFY = 0.50/0.80), SPLS outperforms SPCR by taking advantage of the information included in the outcome. SPCovR, in all conditions, takes advantage of putting some weight on modeling the outcome in the construction of the components.

**Fig. 2:**
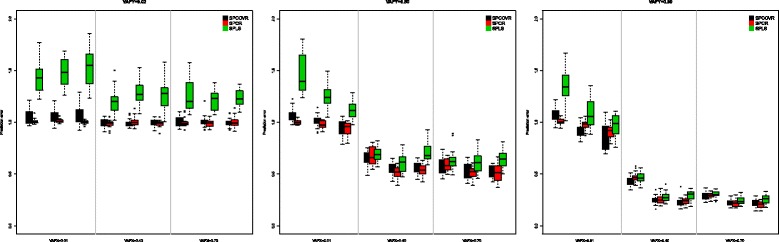
Prediction error for the simulated data

To assess the predictive performance of the methods, the squared prediction error (PRESS) was calculated on test data as follows,

14 $$ PRESS=\frac{\sum_{i} \left(y_{i}-\hat{y_{i}}\right)^{2}}{\sum y_{i}^{2}}  $$

where $\hat {y_{i}}$ was obtained with one of the three considered models and we normalized with respect to the total variation in the observed scores. The lower the PRESS, the better the model performs in terms of prediction. Figure 2 displays the PRESS values for the 27 conditions for each of the three methods. Clearly, as could be expected, when the outcome is submerged in the noise (VAFY = 0.02) all methods perform badly (PRESS ≥ 1). Another striking feature is that SPLS has the largest prediction error in all conditions. When it comes to the relative predictive performance of SPCovR and SPCR in the conditions where the components account for the variation in the outcome (VAFY = 0.50/0.80), the methods seem to perform equally well. Only in the conditions where the components account for almost no variation in the covariates (VAFX = 0.01) but a lot of variation in Y (VAFY = 0.80 and equal strength of the components or more strength of the predictive component) SPCovR outperforms SPCR.

SPCovR was run with three levels of the weighting parameter *α*: namely *α*=0.01, *α*=0.50, and *α*=0.99. For both performance measures and in all conditions, SPCovR with *α*=.99 yields the best results. Hence, it seems that little weight should be given to fitting the outcome in order to obtain good results in terms both of recovering the components and prediction of the outcome. Note that giving no weight at all to the outcome in modeling the components, this is a SPCR analysis, leads to worse recovery in general. For prediction, on the other hand, the gain of using SPCovR is limited to a few conditions and, when the noise in the covariates is considerable, SPCovR is prone to overfitting while SPCR is not.

### Systems biology study of the flu vaccine

We will illustrate SPCovR and compare with SPLS using data that result from a systems biology study on vaccination against influenza [2]. The general aim of this study was to predict vaccine efficacy with micro-array gene expression data obtained soon after vaccination and to gain insight in the underlying biological mechanisms. First we will give a general description of the data and how these were pre-processed, then we wil discuss the SPCovR and SPLS analyses and results.

The authors made data for two seasons, 2007 and 2008, publicly available on the NCBI Gene Expression Omnibus (https://www.ncbi.nlm.nih.gov/geo/) with accession numbers GSE29614 and GSE29617. For both seasons, a micro-array analysis was performed on the genomewide expression in peripheral blood mononuclear cells collected just before and 3 days after vaccination for all participants (26 in 2008 and 9 in 2007). Two different array platforms for measuring gene expression were used but the first 54,675 of 54,715 probe sets of the 2008 season are shared with the 2007 season. Hence, we can use the 2007 data as an independent test sample. Note that the choice for taking the 2007 data as the test set is motivated by the extremely small sample size. The RMA algorithm (Robust Multichip Average; see Irrizary et al. 2003) was used to pre-process the CEL-files. The data collected just before vaccination were considered as the baseline and subtracted from the data at Day 3. For each variable (probeset), the difference scores were centered and scaled to sum-of-squares equal to one. These scaled difference scores form the set of predictor scores **X** in the SPCovR and sparse PLS analyses.

To assess the efficacy of the vaccine, three types of plasma hemagglutination inhibition (HAI) antibody titers were assessed just before and 28 days after vaccination. As described by [2] vaccine efficacy was measured by subtracting the log-transformed antibody titers at baseline from the log-transformed antibody titers 28 days later and taking the maximum of these three baseline-corrected outcomes (to reduce the influence of subjects who started with high antibody concentrations due to previous infection). These maximal change scores were centered, resulting in the scores used as the outcome variable **y** in the SPCovR and sparse PLS analyses.

**Fig. 3:**
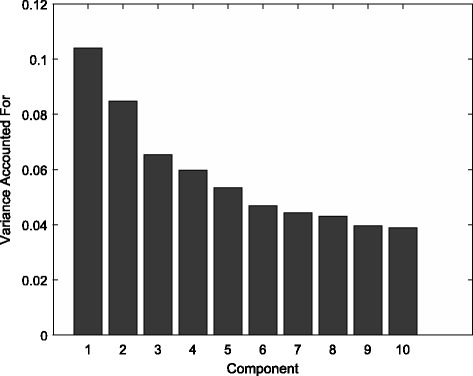
Variance accounted for bye ach PCA component in the predictor data

**Fig. 4:**
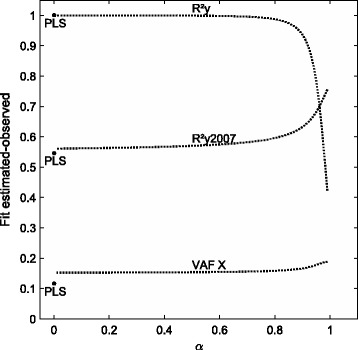
Fit measures obtained for principal covariates regression and partial least square

We start with a principal component analysis of the gene expression data. The variance accounted for by each component is displayed in Fig. 3. We see that the first two components stand out and this will be the number of components that we will use in the PCovR and PLS analyses. To appreciate the flexibility of the weighting of $R^{2}_{X}$ versus $R^{2}_{Y}$ in PCovR, we first consider the non-sparse analyses. The fit measures for the two components resulting from PCovR (with 100 equally spaced values for *α*=.01,.02,…,.99) are compared to those resulting from the PLS analysis using the RGCCA R package [11]: Fig. 4 displays the variance accounted for by the components in the block of predictor variables as well as the squared correlation between observed and modeled outcome scores for the two seasons. As could be expected, the variance accounted for in the block of predictor variables is highest for PCovR with high values of *α*. Because these solutions with high values for *α* give little weight to explaining the variance in the outcome, low $R^{2}_{Y}$ values for the 2008 data are observed. PLS, on the other hand, seems to behave as the other extreme with values similar to those observed for *α*→0. When it comes to the use of the components obtained for the 2008 season to predict the outcome in the 2007 season, better results are obtained with the PCovR components obtained with *α* close to one, this is giving more importance to explaining the variance in the predictor data than in the outcome variable.

Next we turn to the analyses with imposed sparseness on the component weights. The metaparameters of the SPCovR model were set using the proposed stepwise model selection procedure. Hence, based on Fig. 3 a model with two components was selected, *α* was set to a value close to one (*α*=.99), and the ridge penalty was set equal to.05*λ*_1_. In the stability selection procedure, we used *N*=500 resamples, the threshold *π* was set equal to 0.90 and *E*(*V*)=1. This results in *q*_*R*_=416. We compare with the sparse PLS results from two R packages, RGCCA [11] and spls [10] also using *R*=2 components and tuned such that approximately 416 non-zero component weights were obtained in order to have similar sparseness of the sparse PCovR and sparse PLS solutions. spls [10] uses a univariate soft thresholding approach, this is *λ*_2_→*∞*. The SGCCA function in RGCCA [11] was used with the default option for tuning the ridge penalty.

The fit of the solutions to the observed data is summarized in Table 1. The first column shows the variance accounted for by the components in the block of covariates. The SPCovR components account for 19% of the variance while this is much less for the sparse PLS approach as implemented in SGCCA. For the spls package, we could not include such a measure of fit because this package reports fit values only with respect to the outcome variable. On the other hand, the fit of the modeled outcome for the 2008 flu season, which was used to derive the model parameters, is almost perfect for the sparse PLS solutions and low for the sparse PCovR solution. Yet, the predicted antibody titers for the 2007 data, using the estimated component and regression weights of the 2008 analysis, have the highest correlation with the observed antibody titers when the estimates resulting from SPCovR are used ($r(\hat {y}_{2007},y_{2007})^{2}=0.79$ compared to 0.55 and 0.53 for spls and SGCCA respectively).

**Table 1:** Fit of modeled to observed data for three methods: SPCovR, spls, and SGCCA

Method	VAF	$r(\hat {y},y)^{2}$	$r(\hat {y}_{2007},y_{2007})^{2}$
SPCovR	0.19	0.42	0.79
spls		0.99	0.55
SGCCA	0.11	1	0.53

Displayed are the variance accounted for by the components in the block of covariates and the squared correlation between the modeled and observed outcome for the 2008 and 2007 season. The model was constructed using the 2008 data

The percentage of variance accounted for by each of the individual components can be found in Table 2. From these numbers it appears that the first SPCovR component contributes almost exclusively to the variance accounted for in the transcriptomics data while the second component contributes both to the variance accounted for in the transcriptomics data and in the antibody titers. Hence, the first SPCovR component is important for reconstructing the transcription rates in the gene expression data while the second SPCovR component is important both for fitting the transcriptomics data and for predicting the antibody titers. The sparse PLS components resulting from the SGCCA analysis are both focused more towards predicting the antibody titers, the first SGCCA component having the strongest contribution.

**Table 2:** Percentage of variance accounted for in the block of covariates (VAFX) and in the outcome $\left (r(y,\hat {y})^{2}\right)$ by each of the SPCovR and SGCCA components

		Component 1	Component 2
SPCOVR	VAFX	0.10	0.08
	$r(y,\hat {y})^{2}$	0.01	0.40
SGCCA	VAFX	0.07	0.04
	$r(y,\hat {y})^{2}$	0.79	0.20

Another criterion that is important when comparing the different solutions is related to the interpretation of the solution: Do thecomponents reflect a common biological theme that gives insight into the mechanisms that underly vaccines? To answer this question, a functional annotation based on the strength of association of the genes with the components can be performed. The SPCovR and SGCCA results contain such information in two ways, namely in the component weights and in the loadings. The component weights reflect those genes (probesets) that have the strongest contribution to the component scores. Because of the sparseness restriction, only few of them are non-zero. The loadings, on the other hand, reflect the strength of association between the expression values of a particular gene and the component scores. Whereas the component weights measure the unique contribution of a gene on top of the other genes, the loadings measure the strength of association without taking the other genes into account (this is comparable to the interpretation of partial versus univariate correlations); see also [25].

First, we performed a functional annotation of genes associated to the probesets with non-zero component weights using the publicly available annotation tool of PANTHER [26]. A list containing the official gene symbols for the probesets with a non-zero component weight, together with the value of these weights on the two SPCovR and SGCCA components can be found online: https://github.com/katrijnvandeun/SPCovR We performed the functional analysis of these gene lists using the statistical test of over-representation in PANTHER. This means that, for each functional class, the number of genes belonging to that class and present in our list of selected genes was compared to the number of genes for that class in the whole genome. A test of overrepresentation was conducted for each class. An overview of significantly overrepresented functional classes is given in Table 3: Bonferonni correction for multiple testing was used and only classes signficant at the 0.05 level are reported. The first SPCovR component was significantly enriched for rRNA methylation; the second component was significantly enriched for leukocyte activation (and also for its parents, cell activation and immune system process), immune effector process, and negative regulation of metabolic process. Clearly, the second component reflects biological processes thar are important in establishing immunity. This is also the component explaining most of the variance in the outcome and having the highest regression weight: *p*_*y*2_=0.02 compared to *p*_*y*1_=0.004. Notably, the gene encoding for *Calcium/calmodulin-dependent kinase IV (Camk4)* was included as an active predictor in the set. This gene was singled out in the original study of [2] and further validated as an important player in the regulation of the antibody response using knockout experiments. Also, the *BACH2 (Transcription regulator protein BACH2)* gene, which is a known transcription factor necessary for immunity against influenza A virus [27], was included with a very high weight on this component. No significantly over-represented terms were found for the genes underlying the non-zero component weights for the two sparse PLS components obtained with SGCCA. In fact, there was very little overlap in the genes selected by SPLS and SGCCA.Except for one probeset, shared non-zero weights were obtained only between the second SPCovR component and the two SGCCA components. Remarkably, the first SGCCA component is a subset of the second SGCCA component. In the list of non-zero weights (available from https://github.com/katrijnvandeun/SPCovR) it can be seen that only 32 probesets have non-zero weights both for SPCovR and SGCCA, corresponding to 19 unique gene symbols. Relatively high weights in both analyses were obtained for *SMUG1 (Single-strand-selective monofunctional uracil-DNA glycosylase 1)* which has a role in antibody gene diversification and *PPP1R11 (Protein phosphatase 1 regulatory inhibitor subunit 11)* known to effect NF- *κ*B activity [28]. Also for the genes associated to the selected probesets by the sparse PLS analysis performed with the spls package [10], no terms were found.

**Table 3:** Significantly enriched gene ontology classes

Biological process	Nr of genes found	Nr of genes expected	+/−	*P*-value
rRNA methylation	5	.21	+	2.03*E*−02
Cellular macromolecule metabolic process	89	58.65	+	1.68*E*−02
Nucleic acid metabolic process	60	34.14	+	2.84*E*−02
Cellular component organization or biogenesis	75	47.15	+	3.86*E*−02
Gene expression	57	31.88	+	3.30*E*−02
Leukocyte activation	18	4.59	+	6.11*E*−03
Cell activation	20	5.36	+	2.88*E*−04
Immune system process	31	13.13	+	2.79*E*−02
Immune effector process	19	5.25	+	9.41*E*−03
Negative regulation of metabolic process	32	14.16	+	4.59*E*−02

Second, we performed an enrichment analysis based on the loadings. The loadings reflect the strenght of association of a gene with the component with higher loadings indicating that the gene is more important for the process at play. Both PANTHER and GSEA [29] accept as input lists of genes together with a value that indicates the importance of the gene^2^. Output resulting from the enrichment analyses can be found online (https://github.com/katrijnvandeun/SPCovR), here we summarize the main results. A first result of interest is that the same kind of processes are recovered from the enrichment analyses of the loadings as obtained previously when looking for over-represented classes in the gene lists with non-zero component weights. Also here, the annotation of the loadings obtained with SPCovR shows evidence of immune related processes while such evidence is weak for the SGCCA loadings. Notably, some immune related gene ontology terms are found in the enrichment analyses of the SGCCA loadings. In fact, overall more terms are recovered from the enrichment analyses of the loadings. This could be expected given the small number of genes involved in the lists obtained from the non-zero component weights.

Taken together, the results suggest that SPCovR, by putting more emphasis on accounting for the structural variation in the gene expression data when building the prediction model, catches the processes that are important in establishing the immune response to the vaccine. This pays off in the sense that a more stable prediction model is obtained that has better generalizability (and thus better prediction for the held out sample).

## Discussion

Often a large amount of variables is measured with a double goal: Predicting an outcome of interest and obtaining insight in the mechanisms that relate the predicting variables to the outcome. In the high-dimensional setting this comes along with a variable selection problem. Principal covariates regression is a promising tool to reach this double goal; we extended this tool to the high-dimensional setting by introducing a sparse version of the PCovR model and offerering a flexible and efficient estimation procedure.

In this paper we showed through simulation that sparse PCovR can outperform sparse PLS as it allows to put less emphasis on modeling the outcome: By putting more weight on accounting for the variation in the covariates, more insight in the processes that underly the data may be obtained and this, in turn, results in better out-of-sample prediction. The benefit of this was illustrated for publicly available data: clearly a meaningful annotation of the selected genes was obtained with SPCovR while no enriched terms were found for the genes selected by sparse PLS. At the same time, the SPCovR analysis resulted in a much better out-of-sample prediction.

## Appendix

### Derivation of an algorithm for sparse PCovR

Here we will discuss the estimation of the loadings and component weights in the alternating procedure presented in Algorithm 1.

### Conditional estimation of the loadings

Given the component weights, the problem that needs to be solved is to minimize

15 $$\begin{array}{@{}rcl@{}} L(\mathbf{P}_{x},\mathbf{P}_{y}) &=& \big\| \mathbf{Z} - \mathbf{XWP}^{T} \big\|^{2} +\lambda_{1}|\mathbf{W}|_{1} +\lambda_{2}|\mathbf{W}|_{2}^{2}\\ &=& \big\| \mathbf{Z} - \mathbf{TP}^{T} \big\|^{2}+k_{1}, \end{array} $$

such that diag(**P**^*T*^**P**)=**1** (oblique case) or (**P**^*T*^**P**)= **I** (orthogonal case) and with $k=\lambda _{1}|\mathbf {W}|_{1} +\lambda _{2}|\mathbf {W}|_{2}^{2}$ a constant.

This optimization problem can be solved in an iterative procedure that updates each of the loading vectors **p**_*r*_ in turn:

16 $$\begin{array}{@{}rcl@{}} \big\| \mathbf{Z} - \mathbf{TP}^{T} \big\|^{2}+k_{1}&=&  \big\| \left(\mathbf{Z} - \sum_{r\neq r*}\mathbf{t}_{r}\mathbf{p}_{r}^{T}-\mathbf{t}_{r*}\mathbf{p}_{r*}^{T}\right) \big\|^{2}+k_{1}\\ &=& \big\| \mathbf{Q}_{r*}-\mathbf{t}_{r*}\mathbf{p}_{r*}^{T} \big\|^{2}+k_{1} \\ &=& \text{tr}\mathbf{Q}_{r*}^{T}\mathbf{Q}_{r*}- 2\text{tr}\mathbf{Q}_{r*}^{T}\mathbf{t}_{r*}\mathbf{p}_{r*}^{T}\\ &&+ \mathbf{t}_{r*}\mathbf{p}_{r*}^{T}\mathbf{p}_{r*}\mathbf{t}_{r*}^{T}+k_{1}\\ &=& (\text{tr}\mathbf{Q}_{r*}^{T}\mathbf{Q}_{r*}+\mathbf{t}_{r*}^{T}\mathbf{t}_{r*}+k_{1}) \\ &&-2\text{tr}\mathbf{Q}_{r*}^{T}\mathbf{t}_{r*}\mathbf{p}_{r*}^{T}.\\ \end{array} $$

Hence the problem of optimizing each of the **p**_*r*∗_ in turn is equivalent to maximizing $\text {tr}\mathbf {Q}_{r*}^{T}\mathbf {t}_{r*}\mathbf {p}_{r*}^{T}$. The solution to this problem is

17 $$ \mathbf{p}_{r*}^{+}=\frac{\mathbf{Q}_{r*}^{T}\mathbf{t}_{r*}} {\mathbf{t}_{r*}^{T}\mathbf{Q}_{r*}\mathbf{Q}_{r*}^{T}\mathbf{t}_{r*}}.  $$

The solution to the orthogonal case is given by **P**=**VU**^*T*^ with **U** and **V** from the singular value decomposition of **T**^*T*^**Z**. When the number of variables is much larger than the number of observations a more efficient procedure is to calculate the eigen-value decomposition of the *R*×*R* matrix **T**^*T*^**ZZ**^*T*^**T** and to use the resulting eigenvectors and eigenvalues to obtain **VU**^*T*^ (see the implementation for details).

### Conditional estimation of the component weights

Given the loadings, we need to solve the following problem: Minimize with respect to **W**

18 $$\begin{array}{@{}rcl@{}} L(\mathbf{W})&=& \big\| \mathbf{Z} - \mathbf{XWP}^{T} \big\|^{2} +\lambda_{1}|\mathbf{W}|_{1} +\lambda_{2}|\mathbf{W}|_{2}^{2} \\ &=& \big\| \text{vec}(\mathbf{Z})-\text{vec}\left(\mathbf{XWP}^{T}\right) \big\|^{2} \\ &&+\lambda_{1}|\text{vec}(\mathbf{W})|_{1} +\lambda_{2}|\text{vec}(\mathbf{W})|_{2}^{2} \\ &=& \| \text{vec}(\mathbf{Z})-(\mathbf{P}\otimes \mathbf{X})\text{vec}(\mathbf{W}) \|^{2} \\ &&+\lambda_{1}|\text{vec}(\mathbf{W})|_{1} +\lambda_{2}|\text{vec}(\mathbf{W})|_{2}^{2} \end{array} $$

The latter expression can be rewritten as follows:

19 $$ {{}\begin{aligned} L(\mathbf{W}) &= \sum_{i,j} \left(z_{ij}-(\mathbf{p}_{j} \otimes \mathbf{x}_{i})\text{vec}(\mathbf{W}) \right)^{2} \\ & \quad +\lambda_{1}|\text{vec}(\mathbf{W})|_{1} +\lambda_{2}|\text{vec}(\mathbf{W})|_{2}^{2} \\ &= \sum_{i,j} \Big(z_{ij}-\sum_{r} p_{jr}\sum_{j} x_{ij}w_{jr}\Big)^{2} \\ &\quad +\lambda_{1} \sum_{j,r}|w_{jr}| +\lambda_{2} \sum_{j,r}(w)_{jr}^{2}. \\ \end{aligned}}  $$

This is an elastic net regression problem with outcome scores *z*_*ij*_ modeled on the basis of *RJ* variables. To solve the optimization problem, we rely on a coordinate descent procedure [20]: each of the *w*_*jr*_ is updated in turn while keeping the remaining coefficients fixed. Hence, rewriting the loss function with isolation of one specific coefficient *w*_*j*∗*r*∗_, we obtain

20 $$\begin{array}{@{}rcl@{}} &&\sum_{i,j} \left(z_{ij}-\sum_{r} p_{jr}\sum_{j} x_{ij}w_{jr}\right)^{2} \\ &&+\lambda_{1} \sum_{j,r}|w_{jr}| +\lambda_{2} \sum_{j,r}(w)_{jr}^{2} \\ &=&\sum_{i,j} \left(\left(z_{ij}-\sum_{r\neq r*} p_{jr}\sum_{j} x_{ij}w_{jr}\right)-p_{jr*}x_{ij*}w_{j*r*}\right)^{2} \\ &&+\lambda_{1} \sum_{j \neq j*,r \neq r*}|w_{jr}| +\lambda_{1} |w_{j*r*}|\\ &&+\lambda_{2} \sum_{j\neq j*,r\neq r*}(w)_{jr}^{2} + \lambda_{2} w_{j*r*}^{2} \\ &=&\sum_{i,j} \left(r_{ij}-p_{jr*}x_{ij*}w_{j*r*}\right)^{2} \\ &&+k_{2} +\lambda_{1} |w_{j*r*}|+ \lambda_{2} w_{j*r*}^{2}. \\ \end{array} $$

This is a univariate elastic net regression problem with the following solution

21 $$ \left\{ \begin{array}{l} w_{j*r*}^{+} =\frac{\sum_{i,j}r_{ij}p_{jr*}x_{ij*}-\lambda_{1}/2}{\lambda_{2}+ \sum_{i} x_{ij*}^{2}}, \\ \quad \text{if}\ 0<\sum_{i,j}r_{ij}p_{jr*}x_{ij*}<\lambda_{1}/2 \\ \\ w_{j*r*}^{+}=\frac{\sum_{i,j}r_{ij}p_{jr*}x_{ij*}+\lambda_{1}/2}{\lambda_{2}+\sum_{i} x_{ij*}^{2}}, \\ \quad \text{if} -\lambda_{1}/2<\sum_{i,j}r_{ij}p_{jr*}x_{ij*}<0 \\ \\ w_{j*r*}^{+}=0, \text{otherwise}. \end{array}\right.  $$

The procedure may seem slow given the loop over *RJ* coefficients and the involvement of large matrices in the computations. We accounted for these computational issues in our implementation by rewriting the expressions in (21) and making use of the properties of **P**. A further speed up may be obtained by warm restarts and active set learning, this is cycling over the non-zero coefficients only. Further improvement over the GLMnet procedure [20] was obtained by accounting for its dependence on the order of the variables as described in [31].

## Abbreviations

BACH2: Transcription regulator protein BACH2
CAMK4: Calcium/calmodulin-dependent kinase IV
HAI: Hemagglutination inhibition assay
LARS: Least angle regression
PCA: Principal component analysis
PCovR: Principal covariates regression
PCR: Principal components regression
PLS: Partial least squares
PP1R11: Protein phosphatase 1 regulatory inhibitor subunit 11
PRESS: Squared prediction error
RMA: Robust Multichip Average
SMUG1: Single-Strand-Selective Monofunctional Uracil-DNA Glycosylase 1
SPCovR: Sparse principal covariates regression
SPCR: Sparse principal components regression
SPLS: Sparse partial least squares
VAF: Variation accounted for
VAFX: Proportion of variation accounted for in the covariates
VAFY: Proportion of variation accounted for in the outcome

## Footnotes

1 [30] proposed a so-called sparse principal components regression method that in fact is a sparse covariates regression method. As this method, implemented in the spcr R package, gave an out-of-memory failure on the illustrative example we do not consider it further.

2 The reason to also consider GSEA and not only PANTHER is that the latter only allowed to use a very limited set of gene ontology terms in the enrichment analysis, unlike in the overrepresentation analysis.
